# Swallow Strength and Skill Training with biofeedback In acute Post stroke dysphagia (ssSIP): a protocol for a multi-centre feasibility trial

**DOI:** 10.1186/s40814-026-01803-z

**Published:** 2026-03-18

**Authors:** Jacqueline K. Benfield, Kirsten Woods, Lisa Woodhouse, Cristina Roadevin, Marilyn James, Philip M. Bath, Timothy J. England, Kate Radford, Catriona Steele

**Affiliations:** 1 Division of Clinical Neuroscience and Mental Health, School of Medicine, University of Nottingham, Nottingham, United Kingdom; 2 Nottingham Clinical Trials Unit, School of Medicine, University of Nottingham, Nottingham, United Kingdom; 3 Centre for Rehabilitation & Ageing Research, School of Medicine and NIHR Nottingham Biomedical Research Centre, Nottingham, United Kingdom; 4 KITE, Toronto Rehabilitation Institute, University Health Network, Toronto, Canada; 5 Present Address: Derbyshire Community Health Services NHS Trust, DERBY, United Kingdom

**Keywords:** Dysphagia, Rehabilitation, Strength and skill training, Stroke, Dose

## Abstract

**Background:**

Post stroke dysphagia is common and has negative consequences on health and wellbeing. Collectively, behavioural interventions including swallow strength and skill training with biofeedback improve dysphagia, but little is known about which interventions are most effective and at what dose. Early intensive intervention may be most beneficial for motor recovery, but it is unclear whether dysphagia therapy such as this is feasible to deliver in the inpatient acute stage.

**Aim:**

To understand the feasibility of clinical teams delivering swallow strength and skill training with biofeedback in acute inpatient stroke and explore which dose may result in better outcomes.

**Methods:**

One hundred twenty participants from inpatient stroke units 1–8 weeks post stroke in the UK will be recruited in this multi-centre prospective randomised controlled feasibility trial. If eligible, they will be randomised 1:1:1 to either usual care, low-intensity therapy (once daily) or high-intensity therapy (twice daily) groups. In addition to usual care, treatment groups will receive 1 or 2 × 35-min daily sessions of swallow strength and skill training with surface electromyographic biofeedback over 2 weeks. The primary outcome is feasibility measured by recruitment and treatment fidelity. Secondary outcomes will explore treatment effects and dose of the intervention on swallowing, clinical, quality of life and health economic outcomes. Embedded qualitative process evaluation of intervention delivery (fidelity, barriers and enablers), clinical staff training and trial procedures informed by the Conceptual Framework for Intervention Fidelity (CFIF) and Conceptual Framework for Implementation Research (CFIR). Criteria have been outlined for progression to a definitive study.

**Discussion:**

This trial will inform the feasibility of a definitive study to determine the effectiveness of optimal dose swallow strength and skill training with surface electromyographic biofeedback on the severity of dysphagia 3 months post stroke. The process evaluation will inform refinements to the intervention, training and trial procedures to ensure effective delivery.

**Trial registration:**

Registration Clinical Trials.gov NCT05744245, date of release 20/12/2022.

**Supplementary Information:**

The online version contains supplementary material available at 10.1186/s40814-026-01803-z.

## Background

More than 40% of patients with acute stroke have oropharyngeal dysphagia which has negative impacts on health and wellbeing [1–5]. Over 30% of people with acute stroke have ongoing dysphagia at 30 days [6], demonstrating the need for effective interventions to improve dysphagia.

No single intervention has a robust evidence base shown to improve dysphagia, but collectively behavioural interventions may improve swallowing, reduce pneumonia risk and reduce length of stay [7]. One intervention, swallow strength and skill training (ST) with surface electromyography biofeedback (sEMG), aims to improve the amount of force a patient can exert during swallowing by strengthening muscles weakened by stroke (strength training) and improve the control a patient has over the timing and force of their swallow (skill training) to successfully swallow different types of foods and drinks. The Biofeedback in Strength and Skill Training (BiSSkiT) software paired with submental muscle group sEMG can give users visual feedback on the amplitude and timing of their swallow and sets progressively more challenging targets based on user performance [8]. It has been found to be acceptable to patients and feasible to deliver by *researchers* in acute stroke [9]. Little is known about what dose of this, or any behavioural intervention [10, 11] is most effective and whether clinical teams in acute inpatient stroke units are able to implement this intervention.

### Objective

To investigate the feasibility of delivering swallow strength and skill training (ST) by clinical teams in acute inpatient stroke National Health Service (NHS) settings and explore which dose of the intervention results in the best treatment effect for dysphagia severity and may be the most cost-effective.

## Methods

### Design

Swallow Strength and Skill Training with biofeedback In acute Post stroke dysphagia (ssSIP) is a multi-centre, prospective, assessor-blinded, three-arm randomised controlled feasibility trial with embedded process and health economic evaluation.

### Participants

Adults with a new moderate to severe dysphagia will be recruited from inpatient acute stroke and rehabilitation hospitals, from 1 to 8 weeks after a new stroke. Moderate to severe dysphagia is defined as a dysphagia severity rating scale [12] score of ≥4 or safety concerns with thin fluids identified by the clinical speech and language therapist (SLT). Given alertness levels and dysphagia may resolve rapidly in some patients and so therapy is therefore not necessary, we will exclude those that demonstrate this rapid progression. Participants will have sufficient visual, cognitive and communication skills to participate in the intervention which will be verified by a screening test by a trained SLT. This will involve being able to perform a volitional swallow, being able to demonstrate ability to point to their swallow signal on the screen and follow the instructions of the task. Clinical teams will obtain written consent from participants or their proxy if they do not have the capacity to consent.

Participant inclusion criteria:

- Adults over 18
- Clinical diagnosis of stroke
- >1 week and <8 weeks post stroke
- New moderate to severe dysphagia (DSRS ≥ 4) or patients where the SLT has identified safety risks with full amounts of thin level 0 fluids
- Not rapidly improving dysphagia (2 clinical SLT assessments over a week showing minimal change in DSRS)
- Pass an eligibility screen—ability to perform a volitional swallow and have sufficient visual, cognitive and communication skills to participate in the intervention

Participant exclusion criteria:

- Medically unwell (judged by clinical team), Glasgow Coma Scale (GCS) <10, on >4 L oxygen, poor prognosis, end of life care
- Previous dysphagia
- Degenerative neurological condition
- Patient likely to be repatriated to or rehabilitated at another organisation within 10 days
- Participation in another trial aimed at improving dysphagia
- Unwilling to remove beard/hair from under chin

### Approvals and amendments

ssSIP has been approved by the Bromley Research Ethics Committee (Ref: 23/LO/0131) and by local research and innovation teams and is registered on ClinicalTrials.gov (NCT05744245).

A substantial amendment to include participant interviews in the protocol and a non-substantial amendment clarifying inclusion criteria were approved on 18 August 2023 and 29 January 2024 respectively. Submission for publication occurred pre-completion of the trial to ensure transparency, methodological comparability and groundwork for future trials.

### Randomisation, allocation concealment and blinding

Once a participant has consented and has passed the eligibility screening assessment, they will be randomised to one of three groups: ST-0 which is no treatment sessions per day, ST-1 is one treatment session per day and ST-2 is 2 treatment sessions per day. All three groups will continue to receive usual care (UC). Participants will be computer randomised by minimisation on age, stroke severity and dysphagia severity through the secure server of the database. This will ensure allocation concealment. The minimisation rules were age <70/70+, NIHSS total <10/10+ and DSRS total <9/9+ to ensure groups were evenly matched by factors known to impact swallow recovery. To ensure that we retain a random element to treatment allocation, 5% will be randomised by simple randomisation. Outcome assessors will be blinded to group, but participants, therapists and clinical teams will not be blinded given the nature of the intervention.

### Trial procedures

All trial procedures are summarised in Table 1.

**Table 1:** Schedule of enrolment, assessments and interventions

	Screen	Baseline	Days 1–14	Day 15	Discharge/inpatient death	Day 90
Location	Hospital	Hospital	Hospital	Hospital or outside	Hospital	Hospital or outside (phone)
Eligibility	X					
Consent/assent	X					
Eligibility screening assessment		X				
DSRS, FSS, NIHSS Swallow strength and swallow skill		X		X		X
VFS (sub-group) ASPEKT		X		X		
Participant interviews (sub-group)				X		
Randomisation		X				
ST0 vs ST1 vs ST2			X			
Targeted outcomes: pneumonia and number of Abx for LRTI			X	X	X	
All SAEs			X			
Intervention-related (S)AEs			X	X		
Fatal SAEs			X	X	X	X
Usual care data				X	X	
All-cause mortality						X
QoL: EQ-5D-5L, EQ-VAS		X		X		X
mRS, BI, discharge date and location, pneumonia and number of Abx for chest infections, DHI						X
Resource use					X	X

*BI *Barthel index, *mRS *modified Rankin Scale, *DSRS *dysphagia severity rating scale, *DHI *Dysphagia Handicap Index, *EQ-5D-5L *EuroQoL-5-dimension, *EQ-VAS *EuroQoL visual analogue scale, *FSS *Feeding status scale, *NIHSS *National Institutes of Health stroke scale, *QoL *Quality of life, *VFS *Videofluoroscopy, *SAE *Serious adverse events, *LRTI *Lower respiratory tract infection, *ASPEKT *Analysis of Swallowing Physiology—Events, Kinematics and Timing, *Abx *antibiotics

### Intervention

#### Intervention refinement

The intervention protocol has been further refined since the protocol described by Benfield et al. [9]. The refinement process encompassed reviewing the findings from the previous trial, testing the latest technology in collaboration with a public and patient involvement group (PPI) with lived experience of dysphagia and appraising new research literature in neuro-rehabilitation and post stroke dysphagia. The detailed process will be published elsewhere; here we describe the current treatment protocol.

#### Treatment protocol

**Fig. 1:**
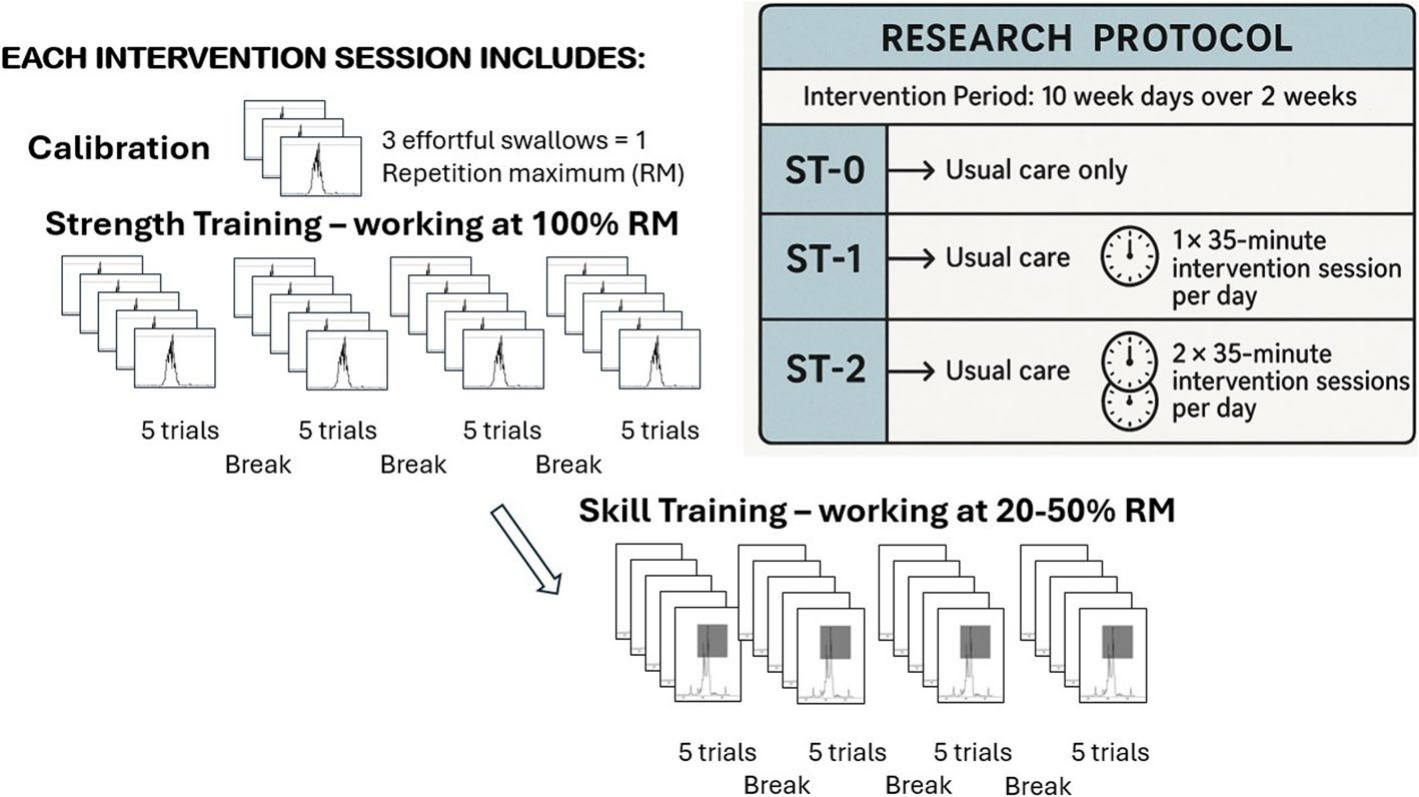
Swallow strength and skill training intervention protocol

Participants receiving the intervention will be set up with electrodes placed on the submental muscles, connected through a surface electromyography device to a laptop. Each therapy session will be 35 min and will involve 40 repetitions of a combination of swallow strength and swallow skill exercises with visual biofeedback about their performance on the laptop screen in front of them. The starting point of the exercises is calibrated to the individual and is based on the mean sEMG amplitude of the three effortful swallows which we refer to as the repetition maximum (RM). In each block of strength exercises, participants will perform five effortful swallows aiming for a visual target at 100% of their calibrated RM. The exercises become more or less challenging depending on performance. Each block of skill exercises involves performing five swallows with different timing and amplitude targets between 20 and 50% of their RM (Fig. 1). Participants will be asked to aim for the peak timing and amplitude of their swallow to fall within a box on the screen. Visual as well as verbal feedback will be given by those facilitating the intervention. Each session will aim for 20 strength and 20 skill repetitions (swallows) with 60-s breaks after each five-trial block but can be tailored to the individual if the tasks are challenging. Those in the ST-1 group will receive one session per day for 10 days and those in the ST-2 group will receive two sessions per day for 10 days. The therapy will be delivered by trained SLTs or SLT assistants (SLTAs) who are trained to deliver dysphagia rehabilitation. If participants are discharged prior to the end of the intervention period, the treatment will stop.

#### Usual care

All participants will receive usual standard care by the stroke team and the speech and language therapy team which usually includes assessment and management recommendations, patient and family education and rehabilitation.

#### Training and delivery co-design

**Fig. 2:**
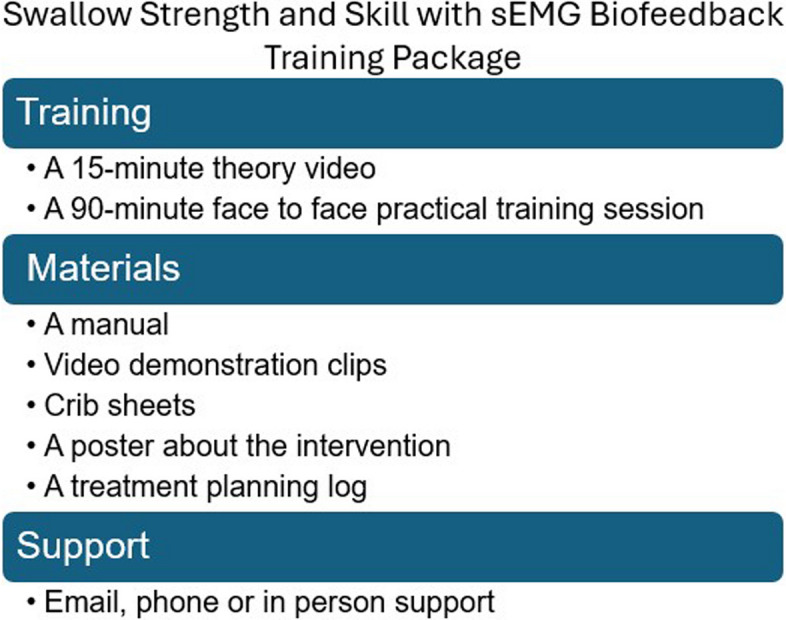
Overview of swallow strength and skill intervention training package

The training package was designed with a group of five SLT(A) stakeholders over a series of online workshops. This also included approaches for implementing high-intensity therapy in the acute inpatient stroke setting. The process will be described elsewhere; here we describe the content of the package (Fig. 2).

#### Training package

An initial cohort of SLT(A)s will receive training comprising a 15-min theory video and a 90-min face to face practical training session including practice setting up and using the equipment. Teams will be given a manual with a brief background, step by step written and visual instructions on how to set up and deliver the intervention and troubleshooting. They will have access to video clips demonstrating setup and delivery of the intervention and crib sheets for use in the therapy sessions to remind them of key steps. A poster about the intervention will also be provided to be displayed on the ward. This aims to spark the interest and support of the multidisciplinary team to help ensure participants are available and ready for therapy sessions. A treatment planning log will also be provided so that SLT(A) teams can plan intervention sessions in advance if required. The resources were designed to enable those that have received the training and have some experience delivering the intervention to train up new colleagues if needed. During the trial, the SLT(A)s will be able to contact the researchers via email or telephone for any further support with any questions or difficulties encountered with adherence to the treatment protocol. They can also request further training including joint treatment sessions with the researchers if there are any uncertainties about intervention delivery. However, there is no formal assessment of therapist competency before participant enrolment.

### Outcomes (including safety and process evaluation)

#### Primary outcome—feasibility

The primary outcome is trial delivery, determined by (a) the trained SLT(A)s ability to deliver the intervention in real-life inpatient acute stroke settings and (b) suitability of trial procedures for use in a future larger-scale trial. An embedded process evaluation will aim to explain qualitative outcomes by exploring treatment fidelity, effectiveness of training and facilitators and barriers to implementation. The following methods will be used to collect data on different aspects of feasibility.

##### Recruitment and retention

The number of participants recruited, the number eligible for the intervention and the number remaining in the trial at the day 90 data collection will all be gathered.

##### Treatment fidelity

Observation sessions with SLT(A)s delivering intervention at the three sites (6 in total) will be conducted by the research team. A fidelity checklist (Additional file 1) will be used to record fidelity based on the prespecified core components of the intervention. Interviews will be conducted with SLT(A)s across different sites who have been trained and have delivered the intervention (description below). Observations and interviews also capture how well the training prepared therapists to deliver the intervention. The number of sessions, length of sessions, repetitions of the exercise per session and deviations from the protocol will be collected after each treatment session by SLT(A)s on case record forms. Usual care data will be collected at day 15 and discharge.

##### Feasibility of implementation

Interviews will be conducted with trial participants and SLT(A)s who delivered the intervention around the feasibility of implementation. Interviews will be semi-structured and iterative; the topic guides (Additional files 2 and 3) will be informed by the Conceptual Framework for Intervention Fidelity (CFIF) [13] and the Conceptual Framework for Implementation Research (CFIR) [14]. The latter also includes the key domains of the COM-B model [15] which is relevant for the participant interviews. These frameworks provide structured, evidence-based constructs that shape the understanding of adherence to and implementation of complex interventions. In addition to informing methods, they will be used to map qualitative findings. Clinical teams will be able to contact the research team for support with intervention delivery during the trial, and the type of problems encountered will be recorded in a request for support log.

##### Feasibility of trial processes

To ensure trial processes are feasible and can be carried forward to a future trial, completeness of case record forms at each follow-up stage and the use of a participant diary will be captured.

Based on previous research [9], progression criteria for taking the trial forward into the next stage are proposed to be:

- At least one participant is recruited per month per site using any necessary strategies required to achieve this.
- Over 80% of the dose is delivered or facilitators are identified to achieve this.
- Over 80% of the core components of the intervention are delivered or facilitators are identified to achieve this.

Table 2 further outlines the progression criteria. Progression would only occur if all criteria are met; otherwise, the intervention and/or trial protocol would require revision.

**Table 2:** ssSIP progression criteria

Area	Objective	Measurement	Criteria
Progression criteria—progress trial if criteria met	There are sufficient numbers of suitable patients for the intervention in acute stroke settings	Number screened vs number eligible for the intervention Number of participants recruited	Proceed: recruitment of >1 patient per site per month and where <1, if strategies to achieve target are identified Stop: if strategies do not achieve target
	SLTs can deliver minimum effective dose of intervention	Case record forms	Proceed: 80% dose is delivered Review: 50–80% Stop: <50%
	The core components can be delivered	Case record forms Observations	Proceed: 80% of core components delivered Review: 50–80% Stop: <50%
Feasibility of trial processes Take forward process if criteria met	Training acceptable and effective	Interviews with clinicians Requests for support and training	Use if >75% of clinicians found the training acceptable and effective Review if <75% but strategies/resources improve acceptability or effectiveness are identified Change if not
	The health economic data collection is feasible	Use of diary to support resource use data collection	Use if >60% of diaries are completed Review if <60% and strategies support collection Change if not
		Resource use case record form completion	Use if >60% of resource use CRFs are complete Review if <40% but strategies used to enable completion of CRF Change if not
		Practicality of gathering cost information	Quantification of costs using local and published sources Identification of resource items and appropriate unit cost sources
	Each of the outcome measures are feasible to include in the trial	Case record form completion	Use if >80% of each outcome measure is collected accurately Review if <40% but strategies Identified to enable completion of CRF Change if not

#### Secondary outcomes

Swallowing, clinical, quality of life and health economic secondary outcomes will be collected to explore treatment effects and cost-effectiveness of different doses of the intervention. If participants are discharged prior to day 15, we will ask sites to collect outcomes over the phone.

##### Dysphagia severity rating scale (DSRS)

The DSRS is a validated tool to grade the severity of swallowing impairment in post stroke dysphagia [12]. It is a 13-point scale, capturing diet and fluid intake and level of supervision required, and higher scores indicate more severe dysphagia. It will be used to determine the treatment effect of the different doses of ST on dysphagia severity at days 15 and 90 and speed of recovery.

##### Swallow strength and skill

Swallow strength is estimated by mean normalised effortful swallow [16] captured by calculating the mean difference between five effortful swallows and five regular swallows using sEMG and the BiSSkiT software. Swallow skill is estimated by percent of accurate attempts at swallowing at a target time and amplitude window on the BiSSkiT software. Similar paradigms have previously been described [17, 18] to capture the ability to modulate swallowing based on peripheral stimuli which is necessary in safe swallowing of food and liquids. Swallow strength and skill assessment will be carried out at baseline and at day 15 to explore treatment effect on swallow strength and skill.

##### Analysis of Swallowing Physiology: Events, Kinematics and Timing (ASPEKT)

Participants at one site will be invited to have a videofluoroscopy (VFS) at baseline and day 15. The assessment protocol will be embedded in the local clinical protocol and will include four comfortable sips of thin fluids (International Dysphagia Diet Standardisation Initiative, IDDSI, level 0), one comfortable sip of level 2 mildly thick fluids and one 5-ml teaspoon of level 4 puree diet. All trial stimuli are prepared to 40% weight to volume EZ HD 98% Bracco UK LTD barium and drinks thickened with Nutilis Clear Nutricia. Continuous images were recorded onto DVD with a Phillips system at a frame rate of 25 fps. Each recording will be spliced into separate boluses and anonymised. Each bolus will be analysed with the ASPEKT method [19] to explore any treatment effects on swallow physiology.

##### Clinical outcomes

Mortality, number of chest infections and length of hospital stay will be captured at day 90.

##### Quality of life; EuroQol 5 Dimension 5 Level Scale (EQ‑5D‑5L) and Dysphagia Handicap Index (DHI)

The EQ-5D-5L [20], a valid and reliable tool for measuring health-related quality of life, will be collected at baseline, day 15 and day 90. The DHI has undergone a degree of validation with mild to moderate dysphagic patients including an unspecified number of stroke patients. It was found to differentiate between controls and those with dysphagia, has high internal validity and test–retest reliability and is sensitive to significant differences in the severity of dysphagia [19, 21]. PPI members felt that participants early post stroke may not have sufficient insight into their dysphagia, so the DHI will be collected at day 90 only.

##### Resource use

Data on resource use will be collected from participant’s notes at day 15 and discharge. On discharge, participants will be given a diary to record any contact with health professionals related to their swallowing up to day 90. On the day 90 follow-up, participants will be asked questions about their health and the services they have accessed since discharge referring to the diary.

##### Safety

The intervention has an excellent safety record (9) but it is important to collect any related adverse events. Therefore, adverse and serious adverse events (SAEs) will be captured from 0 to 15 days, procedure-related SAEs over days 0–15, and discontinuations due to SAEs and fatal SAEs over days 0–90. An AE includes (1) an exacerbation of a pre-existing illness, (2) an increase in frequency or intensity of a pre-existing episodic event or condition, (3) a condition detected or diagnosed after medicinal product administration even though it may have been present prior to the start of the study and (4) a continuous persistent disease or symptoms present at baseline that worsen following the start of the study. An SAE is any adverse event occurring following study mandated procedures, having received the treatment or intervention that results in any of the following outcomes: (1) death, (2) a life-threatening adverse event, (3) inpatient hospitalisation or prolongation of existing hospitalisation and (4) a disability/incapacity.

### Data collection and management

A trial database (REDCap) will be used by local site investigators to enter data in electronic case record forms. The investigators will use the database to collate and organise anonymised data; this ensures secure storage and maximises data quality. Local trial sites will collect and store personal data for participants for 1 year after the trial ends in order to follow up and share findings of the research, after which it will be archived.

### Trial management (including data monitoring)

The trial coordination centre will be responsible for setup and day to day management of the trial. They will also carry out central and remote data monitoring to ensure timely and complete data entry. Remote monitoring will consist of reviewing the site file and checking that source data matches the collected data and trial protocol and procedures have been followed. A proportionate steering/trial management meeting will be held quarterly with co-investigators and two independent members. Progress will be monitored by the committee and decisions made to continue with the trial. A data monitoring appointee will review the data (unblinded to treatment allocation) mid-way through the study to review the scientific merit of the trial and verify there are no related adverse or serious adverse events.

### Sample size

This is a feasibility study and therefore it is not powered to detect a statistically significant intervention effect. Sample size target will be 120; this will allow for a dropout rate of 10% and an estimated 4% who will fail the screening assessment to achieve 34 participants per group for final analyses. Similarly, the VFS is exploratory and not powered, but a pragmatic sample size of 30 (10 per group) will be used. Given the incidence of dysphagia and data from previous trials [9], this recruitment rate of 1–2 per month per site over 24 months should be feasible.

For the clinician and participant interviews, the maximum sample size for each will be 12 due to what is practical for this trial. Fewer participants may be needed to achieve sufficient information power [22] as the aim of the study is narrow, the sample is highly specific to the study, established theoretical frameworks have been used to plan the topic guide and will structure thematic analysis, and the quality of dialogue should be strong as interviewers are experienced SLT(A)s. SLT(A)s will be recruited across all sites, and participants from one site.

### Statistical methods

#### Feasibility and process evaluation data

*Quantitative data*, e.g. intervention session length, number and type of usual care sessions, will be analysed with descriptive statistics using SPSS. Normally distributed data will be presented as means ± SDs; non-normally distributed data as medians (interquartile ranges).

*Qualitative data*, e.g. interviews and observations, will be analysed using a qualitative descriptive approach [23] which involves largely documenting what the participants say and summarising without extensive interpretation. Transcripts will be coded by two separate individuals on the ssSIP trial using N-VIVO. The data collected will be analysed using thematic analysis [24, 25] as a way of identifying, analysing and reporting any meaningful patterns and themes. Data familiarisation, line-by-line coding and development of broad themes will be used. The themes will then be mapped on to the abovementioned CFIR, CFIF and COM-B model framework domains.

#### Exploratory analysis of secondary outcome measures

The trial is not sufficiently powered to formally assess efficacy. However, exploratory analysis will be conducted on an intention to treat basis to compare effect sizes and 95% confidence intervals for each of the treatment groups. Planned analyses are detailed in the Statistical Analysis Plan (Additional file 4).

#### Health economic evaluation

A pilot health economic evaluation will be conducted to test the health economic methods for use in future definitive economic evaluations and feasibility of data collection and sensitivity of measures of health-related quality of life measures.

The primary health economic analysis will adopt an NHS and personal social services cost perspective in alignment with NICE guidance (2022) [26]. Secondary analysis will incorporate a broader societal perspective to account for wider impacts of the stroke dysphagia, such as time lost from paid employment, and effects on families and friends.

Data will be gathered using a purposively designed patient resource proforma, which patients will complete themselves. This tool will collect comprehensive data on all aspects of patient treatment and follow-up, including medication, equipment and supplies, inpatient and outpatient hospital visits, social care, primary and community care, time lost from work and informal care.

Key collection points for resource use data are set for day 15, discharge and day 90. Patient/carer diaries, distributed at hospital discharge, will be trialled to collect more accurate data on resource use, such as healthcare professional input, during data collection on day 90. Intervention-specific costs, including treatment (equipment), staff time and staff training to deliver the intervention, will be recorded using intervention logs completed by the study team.

The outcome measure for the economic evaluation will be the number of QALYs (quality adjusted life years) based on a 90-day time horizon. The EQ-5D-5L questionnaire will be administered at baseline, day 15 and day 90. While a new value set for the EQ-5D-5L is under development, we will apply the EQ-5D-3L value set by using the validated crosswalk mapping approach proposed by van Hout and colleagues [27]. All analyses will be conducted in STATA 18.

An incremental approach will be applied across the three arms, calculating an incremental cost-effectiveness ratio (ICER) and incremental net monetary benefit. Cost-effectiveness acceptability curves (CEACs) will illustrate the probability of effectiveness relative to willingness to pay at the NICE threshold of £20–30k per QALY gained. Sensitivity analyses will examine key cost drivers.

### Dissemination

Trial results will be written up and shared in academic journals, through conference presentations and through webinars for SLT(A)s and patients; further dissemination will be informed by discussions with the PPI group.

## Discussion

National Clinical Guideline for Stroke 2023 [28] and NICE Stroke Rehabilitation guidelines 2023 [29] recommend 45 min a day of speech therapy including swallow therapy for those with post stroke dysphagia; however, there is a lack of evidence for specific interventions or effective dosing of intervention. It is evident from the Sentinel Stroke National Audit Programme (SSNAP) reports that clinical teams across the UK are largely failing to deliver this level in intervention and without the evidence for clinical and cost-effectiveness and required dosing it is difficult to build business cases to improve resources. Based on the predetermined progression criteria, this trial will inform the feasibility of a definitive study to determine the effectiveness of optimal dose swallow strength and skill training with surface electromyographic biofeedback on swallow severity outcomes at 3 months post stroke. The process evaluation will inform refinements to intervention, training and trial procedures to ensure effective delivery.

Delivering stroke rehabilitation such as this in the acute inpatient setting is complex [30]. Factors that add to this complexity are variability in the patient’s medical presentation, functional ability and personal characteristics, clinician knowledge, experience and attitudes as well as structural, environmental and cultural factors surrounding where rehabilitation is delivered. Previous research identified several adaptations to the protocol that were needed to deliver the intervention to this acute inpatient population, such as altering session length and incorporating more breaks [9]. However, little is known about how intervention delivery might differ in different sites or how different SLT(A)s and contexts might impact on this or any dysphagia rehabilitation in the acute inpatient stroke setting. By gathering qualitative data from interviews and observations, barriers and facilitators to its success will be captured, which will be beneficial not only to inform the future directions of this research but also other acute inpatient rehabilitation trials.

Given the demand on resources to deliver this intervention, it not only needs to be clinically but also cost-effective. Exploratory data gathered on treatment effects and costs will help to inform which dose demonstrates signals to warrant further research.

## Supplementary Information

Additional file 1: ssSIP fidelity checklist

Additional file 2: ssSIP interview topic guide

Additional file 3: Participant interview topic guide

Additional file 4: Statistical Analysis Plan

Additional file 5: SPIRIT checklist

## Data Availability

Not applicable.

## Abbreviations

Abx: Antibiotics
AE: Adverse event
ASPEKT: Analysis of Swallowing Physiology: Events, Kinematics and Timing
BI: Barthel index
BiSSkiT: Biofeedback in Strength and Skill Training
CFIF: Conceptual Framework for Intervention Fidelity
CFIR: Conceptual Framework for Implementation Research
CI: Confidence interval
CEACs: Cost-effectiveness acceptability curves
DHI: Dysphagia Handicap Index
DSRS: Dysphagia severity rating scale
EQ5D-5L: EuroQol 5_Dimension Health Questionnaire 5-level
EQ-VAS: EuroQoL visual analogue scale
FSS: Feeding status scale
HRA: Health Research Authority
ICER: Incremental cost-effectiveness ratio
IDDSI: International Dysphagia Diet Standardisation Initiative
ITT: Intention to treat analysis
LRTI: Lower respiratory tract infection
mRS: Modified Rankin Scale
NHS: National Health Service
NIHSS: National Institutes of Health stroke scale
NICE: The National Institute for Health and Care Excellence
NIHR: National Institute of Health Research
PI: Principal investigator
PIS: Participant information sheet
QALY: Quality adjusted life year
QoL: Quality of life
R&D: Research and development
RCT: Randomised controlled trial
REC: Research Ethics Committee
RM: Repetition maximum
SAE: Serious adverse event
SD: Standard deviation
sEMG: Surface electromyography biofeedback
SLT: Speech and language therapists
SLTA: Speech and language therapy assistants
SLT(A)s: Speech and language therapists and assistants
SSNAP: Sentinel Stroke National Audit Programme
ssSIP: Swallow Strength and Skill Training In Post stroke dysphagia
ST: Swallow strength and skill training
TSC: Trial Steering Committee
UC: Usual care
UK: United Kingdom
VFS: Videofluoroscopy
