# Supplementary material for: Swallow Strength and Skill Training with biofeedback In acute Post stroke dysphagia (ssSIP): a protocol for a multi-centre feasibility trial
Source: Pilot Feasibility Stud. 2026 Mar 18;12:66. doi: 10.1186/s40814-026-01803-z (PMC13169879; doi:10.1186/s40814-026-01803-z)
Supplement: Supplementary file 1 — Additional file 1: ssSIP fidelity checklist [file 40814_2026_1803_MOESM1_ESM.docx]

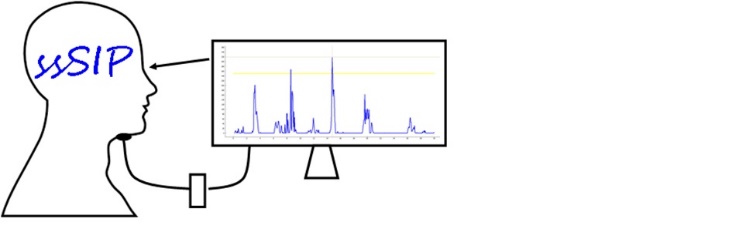


**Swallow Strength and Skill Training with Biofeedback in Acute Post Stroke Dysphagia (ssSIP)**

**Fidelity checklist Version 1.0**

| Site ID | | Clinician ID | | | | Date of observation: | Observer: |
| --- | --- | --- | --- | --- | --- | --- | --- |
| **Component** | | | **Yes/ Done** | **Some extent** | **No/ Not done** | **Barriers, facilitators, comments** | |
| Early intervention (acute phase)  Social and structural context | Patient ready for session | |  |  |  |  | |
|  | Environment – where is it conducted and is it conducive to intervention | |  |  |  |  | |
|  | Other MDT supportive intervention | |  |  |  |  | |
|  | Clinician motivation | |  |  |  |  | |
|  | Participant suitability & motivation | |  |  |  |  | |
| Biofeedback – reliable and clear biofeedback to patients about their performance | Set up | |  |  |  |  | |
|  | Calibration | |  |  |  |  | |
|  | Adequate signal throughout | |  |  |  |  | |
|  | Verbal feedback given | |  |  |  |  | |
|  | Encouragement given | |  |  |  |  | |
| Strength AND skill training | Strength exercises per protocol | |  |  |  |  | |
|  | Strength target achievable | |  |  |  |  | |
|  | Skill exercises per protocol | |  |  |  |  | |
| Exercises are achievable and responsive to patients but provide sufficient challenge | Does the patient match the eligibility criteria in this session? | |  |  |  |  | |
|  | Patient able to achieve 5 trial blocks as per protocol | |  |  |  |  | |
|  | Is the intervention appropriately challenging? | |  |  |  |  | |
|  | Rules followed to step down where challenge is too high | |  |  |  |  | |
| Carried out by trained SLTs and SLTAs | SLT/SLTA trained in dysphagia & ssSIP? | |  |  |  |  | |
|  | Effective set up | |  |  |  |  | |
|  | Appropriate explanation | |  |  |  |  | |
|  | Problem solving & adapting where needed | |  |  |  |  | |
|  | Evidence of knowledge of training | |  |  |  |  | |
|  | Use of training materials | |  |  |  |  | |
|  | Clinician attitude | |  |  |  |  | |
| Therapy is delivered intensively | 40 swallows complete | |  |  |  |  | |
|  | 35-minute session | |  |  |  |  | |
|  | Sessions completed successfully to date | |  |  |  |  | |

Further comments:________________________________________________________________________________________cont. overleaf

__________________________________________________________________________________________________________________

__________________________________________________________________________________________________________________

__________________________________________________________________________________________________________________

__________________________________________________________________________________________________________________

__________________________________________________________________________________________________________________

__________________________________________________________________________________________________________________

__________________________________________________________________________________________________________________

__________________________________________________________________________________________________________________

__________________________________________________________________________________________________________________

__________________________________________________________________________________________________________________

__________________________________________________________________________________________________________________

__________________________________________________________________________________________________________________

__________________________________________________________________________________________________________________

__________________________________________________________________________________________________________________

__________________________________________________________________________________________________________________

__________________________________________________________________________________________________________________

__________________________________________________________________________________________________________________

__________________________________________________________________________________________________________________

__________________________________________________________________________________________________________________

__________________________________________________________________________________________________________________

__________________________________________________________________________________________________________________
