# Supplementary material for: Swallow Strength and Skill Training with biofeedback In acute Post stroke dysphagia (ssSIP): a protocol for a multi-centre feasibility trial
Source: Pilot Feasibility Stud. 2026 Mar 18;12:66. doi: 10.1186/s40814-026-01803-z (PMC13169879; doi:10.1186/s40814-026-01803-z)
Supplement: Supplementary file 2 — Additional file 2: ssSIP interview topic guide [file 40814_2026_1803_MOESM2_ESM.docx]

**
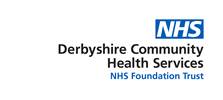

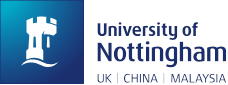
**
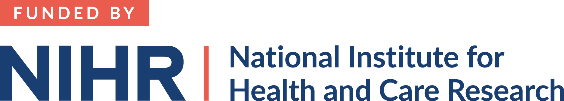


**Interview topic guide: Final Version 1.0**

**3 November 2022**

1. **Demographic information – completed by participant**

Sex?

Ethnicity?

NHS Band?

Years qualified?

Experience in stroke?

What part of the acute pathway do you cover?

Trained by ssSIP team or in house?

1. **Fidelity - Adherence and Moderating factors (CFIF framework)**

**Adherence to the intervention plan**

**Content, Coverage & Quality of delivery (moderator)**

Were you able to deliver the intervention as intended or did you need to make changes or alterations to the intervention so it worked effectively? If so what?

Who benefitted/did not benefit from the intervention? (consider need, capability, opportunity and motivation)

- In what way?

**Moderators**

**Intervention complexity**

How complicated is the intervention?

- Please consider the following aspects of the intervention: duration, scope, intricacy and number of steps involved and whether the intervention reflects a clear departure from previous practices.

**Facilitation Strategies**

**Training**

Do you feel the training prepared you to carry out the roles and responsibilities expected of you? Can you explain?

- What were the positive aspects of the training?
- What was missing?

**Rating** 0-5 usefulness of training

**Materials**

What is your perception of the quality of the supporting materials for implementing the intervention?

- Why?

**Rating** 0-5 usefulness of materials

Support

What supports, such as online resources & phone support, have you accessed?

- When have you accessed these?
- What is your perception of these supports?

**Clinician responsiveness/attitudes**

Do you see value/potential in the intervention?

How does it align and compare to other alternatives that may be considered or that you know about?

- What advantages does the intervention have compared to these other programs?
- What disadvantages does the intervention have compared to these other programs?
- Are there competing priorities?

1. **Social and structural context that may influence intervention implementation and future embedding in practice settings (CFIR framework).**

**Inner setting**

**Structural Characteristics**

What is the context where intervention is delivered (including SLT staffing, ward configuration, number of beds, technology, admissions and LOS)?

What does usual care with regards to dysphagia management usually consist of?

Are there any structural or organisational factors that facilitated or prevented this intervention from being delivered? Consider team configuration, team infrastructure, SLTAs, ward occupancy competing demands, ability to invoice for SLT time etc.)

**Communication and connectedness**

How was the intervention delivery organised between the SLT team? What role did different members of the team hold in delivery/implementation?

How did this work with the wider MDT team?

**Culture**

To what extent are new ideas embraced and used to make improvements in your organization?

- Can you describe a recent example?

How do you think your organization's culture (general beliefs, values, assumptions that people embrace) would affect the continued implementation of this intervention?

**Implementation Climate**

What would be the general level of receptivity in your organization to continue implementing this intervention?

- Why?

**Compatibility**

How compatible is the intervention within your setting and within the existing stroke care pathway?

**Leadership Engagement**

What kind of support or actions have you had from leaders in your organization to help make implementation successful?

- What kind of support can you expect going forward? Can you provide specific examples?
- What types of barriers might they create?

**Available Resources**

Would you expect to have sufficient resources to implement and administer the intervention?

Does the wider team have sufficient resources to support this intervention in this setting?

**Outer setting**

**External Policy & Incentives**

Are there any external factors that might have influenced or would influence implementation of this intervention?

Ask about national level events or changes, NHS pressures, attituded of those outside the organisation guidelines, links to other stroke teams, social media etc.
