# Supplementary material for: Swallow Strength and Skill Training with biofeedback In acute Post stroke dysphagia (ssSIP): a protocol for a multi-centre feasibility trial
Source: Pilot Feasibility Stud. 2026 Mar 18;12:66. doi: 10.1186/s40814-026-01803-z (PMC13169879; doi:10.1186/s40814-026-01803-z)
Supplement: Supplementary file 3 — Additional file 3: Participant interview topic guide [file 40814_2026_1803_MOESM3_ESM.docx]

**Interview topic guide**

A preliminary topic guide has been outlined below:

| Construct name | Overarching question to be answered | Possible probing questions |
| --- | --- | --- |
| **Need**  Insight and understanding of the need for therapy and what therapy is for? (e.g. to stop them coughing, to help them eat and drink normally)  *(The individual(s) has deficits related to survival, well-being, or personal fulfilment, which will be addressed by implementation and/or delivery of the innovation.)* | I wanted to start by talking to you about the changes you’ve noticed since your stroke. Have you noticed any changes with your swallowing? What are you doing to help manage and improve it? | - Are you eating and drinking the same as you normally would? Tell me more about this. - What have the Speech & Language Therapists recommended to help manage your swallowing problem? - Have you been doing any therapy for your swallowing on the ward? - Can you tell me about any of the exercises you have been doing? Can you name any of the exercises or describe them? - Can you tell me what the therapy is for? - Do you know why you should be doing swallowing therapy? Has anyone explained this to you? |
| **Capability**  Psychological/Physical  *(The individual(s) has interpersonal competence, knowledge, and skills to fulfil Role.)* | So we've talked about the swallowing exercises you've been given. Do you understand how to do each of these exercises?  Do you feel like you've got the physical ability to carry each of them out? | - For example, you've been doing X (e.g. CTAR or ST (ssSIP)). Do you understand what you have to do to carry this out? - Do you know the evidence behind the swallowing therapy you are doing? - Have you had the right training? Are you given any feedback on how well you are able to carry out these exercises? - Have you found that easy or difficult to complete? Why? (e.g. too tiring). - Are there some exercises you prefer over the others? Why? - What do you think about the length of the sessions? - What did you think about the frequency of the sessions? |
| **Opportunity**  Social/Physical  *(The individual(s) has availability, scope, and power to fulfil Role.)* | Can you describe what opportunities you have had to do swallowing exercises? | - Is there anything that gets in the way of you doing your exercises? - Is there anything that helps you do your exercises? - Are there adequate opportunities to practice your swallowing exercises? - Would you do more/less if you could? |
| **Motivation**  Automatic/Reflective  *(The individual(s) is committed to fulfilling Role.)* | What part of your swallowing management and therapy do you find motivating? | - For example, you've been doing X. Are you motivated/unmotivated to do this? Do you like/dislike doing X? - Do you believe doing X is useful? - How optimistic or pessimistic are you that doing X has helped with your swallowing? Do you think you’ve seen an improvement? - Do you expect a worthwhile outcome? What is most/least motivating? - Are there other things that you want to achieve that could interfere with doing your swallowing therapy? - How much do you feel you need to do your swallowing therapy? - Are there incentives to do your swallowing therapy? |
