# Supplementary material for: Swallow Strength and Skill Training with biofeedback In acute Post stroke dysphagia (ssSIP): a protocol for a multi-centre feasibility trial
Source: Pilot Feasibility Stud. 2026 Mar 18;12:66. doi: 10.1186/s40814-026-01803-z (PMC13169879; doi:10.1186/s40814-026-01803-z)
Supplement: Supplementary file 4 — Additional file 4: Statistical Analysis Plan [file 40814_2026_1803_MOESM4_ESM.docx]

**Additional File 5 Statistical analysis plan**

**Recruitment data**

Screening and enrolment data will be presented in following way:

FIGURE 1. CONSORT diagram

Screened for eligibility (n= )

## Screened

Excluded (n= )

♦  Reasons not eligible (n= )

Declined to participate (n= )

Consented and completed eligibility screening assessment (n= )

## Enrollment

Excluded (n= )

Vision (n= )

Cognition (n= )

Volitional swallow (n= )

Randomized (n= )

## Allocation

Allocated to **low dose** (n= )

♦ Received allocated intervention (n= )

♦ Did not receive allocated intervention (give reasons) (n= )

Allocated to **high dose** (n= )

♦ Received allocated intervention (n= )

♦ Did not receive allocated intervention (give reasons) (n= )

Allocated to control (n= )

## Follow-Up

Lost to follow-up (give reasons) (n= )

Discontinued intervention (give reasons) (n= )

Lost to follow-up (give reasons) (n= )

Lost to follow-up (give reasons) (n= )

Discontinued intervention (give reasons) (n= )

## Assessment

Assessed for objective 1 (n= )

Assessed for objective 2 (n= )

Assessed for objective 1 (n= )

Assessed for objective 2 (n= )

Assessed for objective 1 (n= )

Assessed for objective 2 (n= ), Etc ...

Etc ..

Feasibility – quantitative data

| Process | Outcome |  |
| --- | --- | --- |
| Eligibility | Number consented |  |
|  | Number failing eligibility screening assessment |  |
| Treatment fidelity | % of core components received |  |
| Training & training materials | No. of requests for support by clinicians |  |
|  | Clinician rating – usefulness of training (/5) |  |
|  | Clinician rating – usefulness of training materials (/5) |  |

Dose of treatment and usual care

| Outcome | | Total | Low dose ST | High dose ST | Control |
| --- | --- | --- | --- | --- | --- |
| Number of ST sessions (mean, SD) | |  |  |  | n/a |
| Length of ST sessions | |  |  |  |  |
| Number of strength trials/session | |  |  |  | n/a |
| Number of skill trials/session | |  |  |  | n/a |
| Number of usual care sessions | |  |  |  |  |
| Length of usual care sessions | |  |  |  |  |
| Type of usual care sessions | Assessment |  |  |  |  |
|  | Therapy |  |  |  |  |
|  | Education |  |  |  |  |

Demographic information

| **Variable** | | **Total**  n= | **Low dose**  n= | **High dose**  n= | **Control**  n= |
| --- | --- | --- | --- | --- | --- |
| Age | | Mean (SD) |  |  |  |
| Sex, female (%) | | Mean (SD) |  |  |  |
| Ethnic group | | N (%) |  |  |  |
| White British etc | |  |  |  |  |
| Previous stroke | | N (%) |  |  |  |
| Premorbid Modified Rankin Scale (/6) | | Median (IQR) |  |  |  |
| Days to randomisation post stroke | | Mean (SD) |  |  |  |
| Stroke type | Haemorrhagic | N (%) |  |  |  |
|  | Ischaemic | N (%) |  |  |  |
| Stroke syndrome | TACS | N (%) |  |  |  |
|  | PACS | N (%) |  |  |  |
|  | POCS | N (%) |  |  |  |
|  | LACS | N (%) |  |  |  |
| CT evidence of atrophy or small vessel disease | | N (%) |  |  |  |
| NIHSS (/42) | | Mean (SD) |  |  |  |
| MDT documented: | |  |  |  |  |
| Visual impairment | | N (%) |  |  |  |
| Dysarthria | | N (%) |  |  |  |
| Aphasia | | N (%) |  |  |  |
| Dyspraxia | | N (%) |  |  |  |
| Cognitive impairment | | N (%) |  |  |  |
| Sensory impairment | | N (%) |  |  |  |
| Dysphagia severity rating scale (/12) | | Median (IQR) |  |  |  |
| Feeding Status Scale | Oral diet - normal | N (%) |  |  |  |
|  | Oral diet - modified | N (%) |  |  |  |
|  | NG feeding | N (%) |  |  |  |
|  | PEG feeding | N (%) |  |  |  |
|  | Other | N (%) |  |  |  |

Analysis of secondary outcomes

| Variable | | Total  n= | Low dose ST  n= | High dose ST  n= | Control  n= | MD/OR (95% confidence intervals) |
| --- | --- | --- | --- | --- | --- | --- |
| Day 15 | |  |  |  |  |  |
| Dysphagia severity (DSRS) (/12) | | Median [IQR] | Median [IQR] | Median [IQR] | Median [IQR] |  |
| Presence of dysphagia (DSRS>0) | | N (%) | N (%) | N (%) | N (%) |  |
| Feeding Status Scale (FSS) (/6) | | Median [IQR] | Median [IQR] | Median [IQR] | Median [IQR] |  |
| NG/PEG tube | | N (%) | N (%) | N (%) | N (%) |  |
| Swallow strength (mean % increase in amplitude between normal and effortful swallows), | | Mean (SD) | Mean (SD) | Mean (SD) | Mean (SD) |  |
| Swallow skill (% of correct timing targets) (/5) | | Median [IQR] | Median [IQR] | Median [IQR] | Median [IQR] |  |
| LRTI & pneumonia | | N (%) | N (%) | N (%) | N (%) |  |
| Antibiotic use | | N (%) | N (%) | N (%) | N (%) |  |
| EQ5D-5L | | Median [IQR] | Median [IQR] | Median [IQR] | Median [IQR] |  |
| EQ-VAS | | Mean (SD) | Mean (SD) | Mean (SD) | Mean (SD) |  |
| Serious adverse events | | N (%) | N (%) | N (%) | N (%) |  |
| Day 90 | |  |  |  |  |  |
| Dysphagia severity (DSRS) | | Median [IQR] | Median [IQR] | Median [IQR] | Median [IQR] |  |
| Presence of dysphagia (DSRS>0) | | N (%) | N (%) | N (%) | N (%) |  |
| Feeding Status Scale | | Median [IQR] | Median [IQR] | Median [IQR] | Median [IQR] |  |
| Dysphagia Handicap Index | | Mean (SD) | Mean (SD) | Mean (SD) | Mean (SD) |  |
| Presence of NG/PEG | | N (%) | N (%) | N (%) | N (%) |  |
| mRS | | Median [IQR] | Median [IQR] | Median [IQR] | Median [IQR] |  |
| Barthel Index | | Mean (SD) | Mean (SD) | Mean (SD) | Mean (SD) |  |
| EQ5D-5L | | Median [IQR] | Median [IQR] | Median [IQR] | Median [IQR] |  |
| EQ-VAS | | Mean (SD) | Mean (SD) | Mean (SD) | Mean (SD) |  |
| Length of stay | | Mean (SD) | Mean (SD) | Mean (SD) | Mean (SD) |  |
| Number of participants with LRTI or pneumonia | | N (%) | N (%) | N (%) | N (%) |  |
| Number of LRTI or pneumonia | | N (%) | N (%) | N (%) | N (%) |  |
| Death | | N (%) | N (%) | N (%) | N (%) |  |
| Discharge destination | 1.Home | N (%) | n1 (%) | n2 (%) | n3(%) |  |
|  | 2.Residential home | N (%) | n1 (%) | n2 (%) | n3 (%) |  |
|  | 3.Nursing home | N (%) | n1 (%) | n2 (%) | n3(%) |  |
|  | 4.Remains inpatient | N (%) | n1 (%) | n2 (%) | n3 (%) |  |

Notes:

A worst score will be assigned at day 90 for people who die (e.g. DSRS=13, FOIS=0) to avoid losing participants in analyses and missing a “kill or cure” effect, and to anchor analyses, as has been done in ENOS, TARDIS and RIGHT-2.

Absolute and relative measures of effect and 95% confidence intervals will be presented for each analysis.

Further exploration of dose response will be conducted for all participants using minutes of therapy received. We will calculate odds ratios using univariate and multivariate ordinal logistic regression adjusting for baseline measures. Finaly time to recovery will be compared across groups using Kaplan Myers plots. For those that didn’t recover a cut off of days to recover will be set at 110 days.
